# Draft Genome Sequences of Two Fusobacterium necrophorum Strains Isolated from the Uterus of Dairy Cows with Metritis

**DOI:** 10.1128/MRA.00201-19

**Published:** 2019-04-25

**Authors:** Amye M. Francis, Soo Jin Jeon, Federico Cunha, KwangCheol Casey Jeong, Klibs N. Galvão

**Affiliations:** a Department of Animal Sciences, University of Florida, Gainesville, Florida, USA; b Department of Large Animal Clinical Sciences, College of Veterinary Medicine, University of Florida, Gainesville, Florida, USA; c D. H. Barron Reproductive and Perinatal Biology Research Program, University of Florida, Gainesville, Florida, USA; University of Arizona

## Abstract

A commensal in the gastrointestinal tract, Fusobacterium necrophorum is involved in the pathogenicity of abscesses, foot rot, and metritis in cattle. Here, we present the draft genomes of two Fusobacterium necrophorum isolates from the uterus of dairy cows with metritis to allow for future comparative genome studies.

## ANNOUNCEMENT

Fusobacterium necrophorum is one of the predominant bacteria associated with uterine infection in dairy cows, being found in greater abundance in metritic cows than in healthy cows (1, 2). F. necrophorum is a Gram-negative non-spore-forming anaerobic rod that originates from the gastrointestinal tract (3), but when it enters other tissues, it can become an opportunistic pathogen that causes necrotic diseases (3). F. necrophorum isolated from liver abscesses of cattle was shown to possess several virulence factors, such as adhesins, leukotoxins, endotoxins, hemolysins, hemagglutinins, and proteases (3–5). However, the mechanisms by which they colonize the uterus and contribute to uterine disease remain unknown.

Swab samples were collected from the uterus of dairy cows in June of 2016 from the University of Florida’s Dairy Research Unit in Hague, FL. Swab samples were transported to the laboratory within 2 hours, and once in the laboratory, swabs were immediately streaked onto Wilkins-Chalgren anaerobe agar (5% horse blood, vitamin K, hemin, Gram-negative spore anaerobic supplement, and kanamycin) and incubated for 48 hours under anaerobic conditions in a BD GasPak system (reference no. 260683) at 37°C. Isolates were again grown on Wilkins-Chalgren anaerobe agar in pure culture, and an agar plate with a pure culture was submitted to Genewiz (South Plainfield, NJ) for Sanger sequencing of the 16S rRNA gene. The taxonomic classification was confirmed by entering the 16S rRNA sequences into NCBI’s standard nucleotide BLAST search using default parameters. The 16S rRNA sequences showed a 99% similarity to the species Fusobacterium necrophorum. Two isolates confirmed as F. necrophorum were named KG34 and KG35. F. necrophorum KG34 and KG35 isolates picked and grown on Wilkins-Chalgren anaerobe agar plates were used for genomic DNA extraction using a QIAamp DNA minikit (Qiagen, Valencia, CA) following the manufacturer’s directions. An Illumina MiSeq platform was used to carry out whole-genome sequencing, and a 2 × 250-bp 500-cycle cartridge was used. The total number of reads for KG34 was 932,174, and the total number of reads for KG35 was 1,093,012. Quality control and trimming of forward and reverse reads for both strains KG34 and KG35 were performed using Sickle version 1.33.1 (6) with a length parameter of 50 and a quality score of 30. The genome was assembled using SPAdes version 3.11.1 (7), and the k-mers used were 21, 33, 55, 77, 99, and 127. Contigs were downloaded from SPAdes and annotated using the NCBI Prokaryotic Genome Annotation Pipeline (PGAP) (8). Default parameters were used for all software. KG34 had a total genome size of 2,051,638 bp, 123 contigs, and a G+C content of 35.25%. The total genome size of KG35 was 2,080,648 bp with 124 contigs and a G+C content of 35.02%. Additionally, KG34 had 2,087 coding DNA sequences (CDSs), and KG35 had 2,084 CDSs. The annotated genomes were searched for known virulence factors of F. necrophorum listed in the literature (3, 9). Proteins of interest in KG34 and KG35 that are potentially virulent included leukotoxin family filamentous adhesion, a hemolysin protein and hemolysin secretion/activation protein, a filamentous hemagglutinin N-terminal domain-containing protein, Clp and omptin proteases, ecotin, a hemin receptor, and a YihY virulence factor. The KG34 proteome also coded for an adhesion protein, and KG35 possessed the virulence-associated protein E. This is the first report of genome sequences of F. necrophorum strains recovered from the uterus of dairy cows with metritis. This report will allow for future comparative genome analysis among F. necrophorum strains that are commensal in the gastrointestinal tract or that cause disease in other body sites or in other species.

### Data availability.

This whole-genome shotgun project has been deposited in DDBJ/ENA/GenBank under the accession no. SBAO00000000 (KG34) and SBAP00000000 (KG35). The versions described in this paper are the first versions, SBAO01000000 (KG34) and SBAP01000000 (KG35). This project and the trimmed reads have been uploaded into the NCBI Sequence Read Archive and can be found under the accession no. SRX5402669 (KG34) and SRX5402668 (KG35).

## References

[1] Santos TMA, Gilbert RO, Bicalho RC 2011 Metagenomic analysis of the uterine bacterial microbiota in healthy and metritic postpartum dairy cows. J Dairy Sci 94:291–302. doi:10.3168/jds.2010-3668. 21183039

[2] Cunha F, Jeon SJ, Daetz R, Vieira-Neto A, Laporta J, Jeong KC, Barbet AF, Risco CA, Galvão KN 2018 Quantifying known and emerging uterine pathogens, and evaluating their association with metritis and fever in dairy cows. Theriogenology 114:25–33. doi:10.1016/j.theriogenology.2018.03.016. 29574306

[3] Nagaraja TG, Narayanan SK, Stewart GC, Chengappa MM 2005 Fusobacterium necrophorum infections in animals: pathogenesis and pathogenic mechanisms. Anaerobe 11:239–246. doi:10.1016/j.anaerobe.2005.01.007. 16701574

[4] Kumar A, Gart E, Nagaraja TG, Narayanan S 2013 Adhesion of Fusobacterium necrophorum to bovine endothelial cells is mediated by outer membrane proteins. Vet Microbiol 162:813–818. doi:10.1016/j.vetmic.2012.10.022. 23153522

[5] Kumar A, Menon S, Nagaraja TG, Narayanan S 2015 Identification of an outer membrane protein of Fusobacterium necrophorum subsp. necrophorum that binds with high affinity to bovine endothelial cells. Vet Microbiol 176:196–201. doi:10.1016/j.vetmic.2014.12.015. 25601800

[6] Joshi N, Fass JN 2011 Sickle: a sliding-window, adaptive, quality-based trimming tool for FastQ files (version 1.33). https://github.com/najoshi/sickle.

[7] Bankevich A, Nurk S, Antipov D, Gurevich AA, Dvorkin M, Kulikov AS, Lesin VM, Nikolenko SI, Pham S, Prjibelski AD, Pyshkin AV, Sirotkin AV, Vyahhi N, Tesler G, Alekseyev MA, Pevzner PA 2012 SPAdes: a new genome assembly algorithm and its applications to single-cell sequencing. J Comput Biol 19:455–477. doi:10.1089/cmb.2012.0021. 22506599 PMC3342519

[8] Tatusova T, DiCuccio M, Badretdin A, Chetvernin V, Nawrocki EP, Zaslavsky L, Lomsadze A, Pruitt KD, Borodovsky M, Ostell J 2016 NCBI Prokaryotic Genome Annotation Pipeline. Nucleic Acids Res 44:6614–6624. doi:10.1093/nar/gkw569. 27342282 PMC5001611

[9] Wright K 2016 Genomics and virulence factors of Fusobacterium necrophorum. PhD thesis. University of Westminister, London, United Kingdom https://westminsterresearch.westminster.ac.uk/item/9ywy3/genomics-and-virulence-factors-of-fusobacterium-necrophorum.

